# ﻿*Mallotus thinii* (Euphorbiaceae), a new species discovered from the coastal areas of the south-central provinces of Vietnam

**DOI:** 10.3897/phytokeys.265.166360

**Published:** 2025-10-31

**Authors:** Nguyen Thi Kim Thanh, Nguyen Diep Anh, Tran Thi Thuy Anh, La Thi Thuy, Nguyen Anh Duc, Tran Duc Long, Van-Son Dang

**Affiliations:** 1 Faculty of Biology, University of Science, Vietnam National University, Hanoi, 334 Nguyen Trai Street, Thanh Xuan Ward, Hanoi, Vietnam University of Science, Vietnam National University Hanoi Vietnam; 2 Institute of Life Sciences, Vietnam Academy of Science and Technology, 85 Tran Quoc Toan Street, Xuan Hoa Ward, Ho Chi Minh City, Vietnam Institute of Life Sciences, Vietnam Academy of Science and Technology Ho Chi Minh Vietnam

**Keywords:** Botanical inventory, Indochina, Khanh Hoa, Quang Ngai, taxonomy

## Abstract

*Mallotus thinii* Kim Thanh & V.S.Dang, a new species discovered in coastal areas of the south-central provinces of Vietnam, is described and illustrated here. This species is morphologically most similar to *M. leptostachyus* Hook.f. and *M. peltatus* (Geiseler) Müll.Arg. It can be distinguished from other *Mallotus* species previously known by its habitat on sandy soils close to the beach, shrubby habit, deciduous stipules, oblanceolate leaf blades, two larger extrafloral nectaries near the base or sometimes two to four more along the midrib, presence of a pistillode, and fruits without spines. A detailed description, color photographs, distribution, habitat, preliminary conservation assessment, and phylogenetic analysis are provided. Molecular phylogenetic analyses based on *matK* and ITS nucleotide sequences strongly confirm *M. thinii* as a distinct new species within *Mallotus*.

## ﻿Introduction

*Mallotus* Lour. is a large genus of Euphorbiaceae, including about 150 species of trees and shrubs that are mainly found in tropical and subtropical regions in Asia, along with a few species in tropical Africa and Madagascar (Sierra et al. 2010). The genus was described by Loureiro (1790) with the type species *Mallotus cochinchinensis* (= *Mallotus paniculatus* (Lam.) Müll.Arg.). After a revision by Pax and Hoffmann (1914) with 10 sections and then Airy Shaw (1968) with eight sections, recent morphological and molecular studies by Sierra et al. (2010) suggested that *Mallotus* s. str. was polyphyletic, in which Mallotus sect. Mallotus, sect. Polyadenii Pax & K.Hoffm., and sect. Stylanthus Pax & K.Hoffm. are monophyletic, while sect. Axenfeldia (Baill.) Pax & K.Hoffm. and sect. Rottleropsis Müll.Arg. are polyphyletic, and sect. Philippinenses Pax & K.Hoffm. is paraphyletic.

In the recent taxonomic revision of Euphorbiaceae for Vietnam, Thin (2007) reported that *Mallotus* s. l. comprised 33 species (excluding the recently synonymized *M. oblongifolius*), two subgenera (*Coelodiscus* (Baill.) Thin and *Mallotus* Müll.Arg.), and six sections (*Axenfeldia* (Baill.) Pax & K.Hoffm., *Hancea* Pax & K.Hoffm., *Rottleropsis* Müll.Arg., *Philippinenses* Pax & K.Hoffm. (former sect. Rottlera), *Stylanthus* Pax & K.Hoffm., and *Mallotus* Pax & K.Hoffm.). Three new species, *Mallotus phongnhaensis* Thin & Kim Thanh, *M. ninhthuanensis* V.S.Dang, Bao & Tagane, and *M. vinhhyensis* V.S.Dang, Tagane & Tk.Yamam., and two new records, *M. leptostachyus* Hook.f. and *M. cordatifolius* Slik, have been reported, increasing the total number of species in Vietnam to 38 (Nguyen and Nguyen 2008; Nguyen et al. 2010; Nguyen and Nguyen 2014; Dang et al. 2025).

In 2007, Nguyen Nghia Thin of VNU University of Science collected unknown *Mallotus* specimens from Binh Dinh Province and deposited them in the HNU Herbarium. Then, in 2018, during a botanical survey conducted in the south-central provinces of Vietnam, the authors discovered wild-growing individuals of this species. The plants, including both male and female individuals, were found along the hedges of local houses and scattered within a nearby area along Sa Huynh Beach, Quang Ngai Province. After six years, in 2023, one more population was discovered along the roadside on Binh Ba Island, Khanh Hoa Province. After morphologically comparing the available herbarium specimens and reviewing relevant literature, these specimens did not match any previously described species. The results led to the conclusion that it was an entirely new species, described here as *Mallotus thinii* Kim Thanh & V.S.Dang.

## ﻿Materials and methods

### ﻿Morphology

The specimens were compared with similar species through a review of taxonomic literature from Vietnam and neighboring areas in Asia (Gagnepain 1925; Ho 1999; Thin 2007; Kiu and Gilbert 2008; van Welzen and Chayamarit 2020) and by examining dried specimens from Vietnamese herbaria (e.g., HNU, NIMM, HN, and VNM) and online digitized images of type specimens available at herbaria such as K and P and websites including Tropicos (https://www.tropicos.org/), Chinese Virtual Herbarium (https://www.cvh.ac.cn/), POWO (https://powo.science.kew.org/), and Asian Plant (https://asianplant.net/).

Measurements with an accuracy of 0.5 mm and descriptions are based on fresh and dried material. The scientific names and terminology follow Turland et al. (2018) (Shenzhen Code), Sierra et al. (2005), and Beentje (2010). Color photographs and images of dried specimens were captured using an Olympus Tough TG-5 camera. Samples for micromorphological details, including pistillate and staminate flowers, pistillodes, and stamens, were fixed and then submerged in water for imaging with a Nikon Z6ii camera and a Nikkor 180 mm f/2.8 AIS lens coupled with a reversed Nikkor AF 50 mm f/1.8D lens. Focus stacking (Helicon Focus) was applied to optimize the micromorphological images.

The conservation assessment is based on the recommendations of the *Guidelines for Using the IUCN Red List Categories and Criteria* (2024).

### ﻿Taxon sampling and DNA analysis

The taxon sampling included 47 sequence-available species of *Mallotus*, nine individuals of the new species, and *Macaranga tanarius* as an outgroup. Taxon names, voucher information, and GenBank accession numbers of the samples used in this study are listed in Table 1 and Table 2. The genetic markers *matK* and ITS were selected based on their previous use and informativeness in earlier studies (Sierra et al. 2010).

**Table 1.** GenBank accession numbers of reference sequences (- sequences not available).

No.	Taxon sampled	*matK*	ITS
1	*Mallotus apelta*	KP093309.1, KP093310.1	KP092931.1, KP092932.1
2	*M. aureopunctatus*	–	MG762732.1
3	*M. barbatus*	EF582633.1	DQ866591.1, KP092933.1
4	*M. brachythyrsus*	EF582634.1	DQ866592.1
5	*M. caudatus*	EF582636.1	DQ866593.1
6	*M. claoxyloides*	EF582639.1	DQ866594.1
7	*M. connatus*	EF582640.1	–
8	*M. cumingii*	EF582642.1	DQ866625.1
9	*M. decipiens*	EF582644.1	DQ866595.1, DQ866596.1
10	*M. discolor*	EF582645.1	DQ866597.1
11	*M. eucaustus*	–	DQ866598.1
12	*M. ficifolius*	EF582647.1	DQ866599.1
13	*M. floribundus*	–	AJ275677.1
14	*M. garrettii*	KR531147	KR532328.1, KR532329.1
15	*M. glabriusculus*	AB924698.1	–
16	*M. griffithianus*	LC737079.1	DQ866600.1
17	*M. hookerianus*	KP093997.1, KP093998.1	KP092934.1, KP092935.1
18	*M. japonicus*	AB268027.1, EF582649.1	MT444827.1, MT444828.1
19	*M. khasianus*	EF582650.1	DQ866601.1
20	*M. korthalsii*	EF582651.1, LC737080.1	–
21	*M. lackeyi*	EF582652.1	AJ298261.1, DQ866602.1
22	*M. leptophyllus*	LC737081.1	–
23	*M. leucocalyx*	EF582654.1	DQ866603.1
24	*M. macrostachyus*	EF582656.1	DQ866604.1
25	*M. miquelianus*	EF582661.1	DQ866605.1
26	*M. mollissimus*	EF582662.1, LK021464.1	–
27	*M. nanus*	AB925120.1	–
28	*M. nudiflorus*	EF582667.1, EF582668.1	DQ866627.1, DQ866628.1
29	*M. oppositifolius*	EF582669.1	DQ866606.1
30	*M. pallidus*	EF582670.1	DQ866607.1
31	*M. paniculatus*	EF582671.1, KP093433.1	DQ866608.1, DQ866609.1
32	*M. peltatus*	EF582672.1, MN885802.1	DQ866610.1
33	*M. penangensis*	–	DQ866611.1
34	*M. philippensis*	EF582674.1, KP093510.1	DQ866614.1, KP092939.1
35	*M. pierrei*	EF582675.1	DQ866615.1
36	*M. pleiogynus*	EF582676.1	–
37	*M. polyadenos*	EF582677.1	DQ866616.1
38	*M. repandus*	EF582678.1, LC506375.1	DQ813305.1, DQ866617.1
39	*M. resinosus*	EF582679.1	DQ866618.1
40	*M. rhamnifolius*	EF582680.1	DQ866619.1
41	*M. rufidulus*	–	DQ866620.1
42	*M. spinulosus*	–	DQ866532.1
43	*M. subpeltatus*	–	DQ866621.1
44	*M. subulatus*	–	DQ866622.1
45	*M. tetracoccus*	EF582683.1	MG762734.1
46	*M. thorelii*	–	DQ866624.1
47	*M. tokiae*	LC498618.1	LC498619.1
48	*Macaranga tanarius*	EF582630.1	DQ866585.1

**Table 2.** Voucher information and GenBank accession numbers of nine individuals sequenced of the new species, *Mallotus thinii*.

No.	Voucher specimens	Locality	GenBank accession numbers	
			*matK*	ITS
1	KT230507-03	Vietnam, Quang Ngai Province, Sa Huynh Commune	PV658520	PV920206
2	KT230507-05		PV658521	PV920207
3	KT230507-06		PV658522	PV920208
4	KT230507-07		PV658523	PV920209
5	KT230507-08		PV658524	PV920210
6	KT230507-10		PV658525	PV920211
7	KT230507-11		PV658526	PV920212
8	KT230507-12		PV658527	PV920213
9	KT230830-04	Vietnam, Khanh Hoa Province, Nam Cam Ranh Commune, Binh Ba Island	PV658528	PV920214

Total DNA was extracted from leaf tissue using an SDS-based protocol (Liu et al. 2000). Amplifications were carried out in 30 µl reactions containing PCR Mastermix 2× (Vazyme Biotech, China), 0.4 µM of each primer, 1.2 µl template DNA, and nuclease-free water. The *matK* or *ITS* region was amplified using primer pairs 5′TCAAATCCTTCGCTATTGGG3′/5′GCGAAATAGAAGAAACTCTTGG3′ or 5′GTAACAAGGTTTCCGTAGGTG3′/5′TGATATGCTTAAACTCAGCGG3′, respectively. The PCR program consisted of an initial denaturation for 5 min at 95 °C, followed by 35 cycles of 20 s denaturation at 95 °C, 20 s annealing at 50 °C, and 45 s extension at 72 °C, with a final extension of 3 min at 72 °C. Sanger sequencing was performed by the 1^st^ Base Company (Selangor, Malaysia). Chromatograms were checked using SnapGene Viewer, and unreliable nucleotide reads were trimmed.

Multiple sequences of each marker were aligned using the ClustalW multiple alignment tool integrated in BioEdit (Hall 1999) with default settings. Sequence ends were manually trimmed, and the two markers were concatenated into a single alignment. The p-distance among samples was estimated using MEGA 11 (Tamura et al. 2021). Substitution models were analyzed with jModelTest 2 (Darriba et al. 2012) for each marker. Based on the BIC score, GTR was selected for *matK* and GTR+I+G for ITS. Phylogenetic trees were reconstructed using matK, ITS, and combined *matK*+ITS sequences through Bayesian inference (BI) in MrBayes v3.2 (Ronquist et al. 2012) under default parameters. For each analysis, two independent runs were performed for 20,000,000 generations, with sampling every 500 generations. Chains, temperature, number of branch swaps, and swap frequencies were set to default. Trees were sampled every 500 generations, the initial 25% of sampled trees were discarded as burn-in, and the resulting trees were visualized using FigTree (v1.4.4).

## ﻿Taxonomic treatment

### ﻿ Mallotus thinii

Taxon classificationPlantaeMalpighialesEuphorbiaceae

Kim Thanh & V.S.Dang sp. nov.

A8FFA032-04C1-5EAE-B011-57C7E101BEB7

urn:lsid:ipni.org:names:77371268-1

Figs 1, 2

#### Type.

Vietnam • Quang Ngai province, Sa Huynh commune, on sandy soils, at the hedge of local people’s houses, 100 meters from the beach, close to Ganh Mountain, 14°38'12.1"N, 109°04'01.4"E, 07 May 2023, *Kim Thanh KT230507-03* (holotype: HNU024793!).

**Figure 1.**
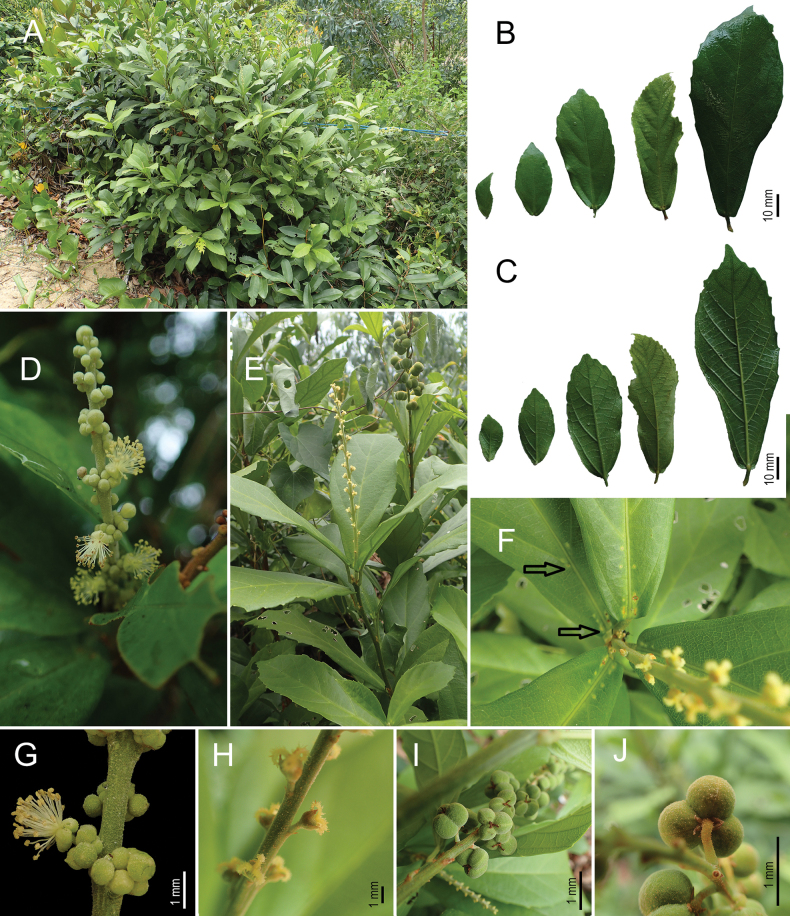
*Mallotus thinii* Kim Thanh & V.S.Dang. A. Habit of new species at the type locality; B. Leaves adaxial surface; C. Leaves abaxial surface; D. Staminate inflorescence; E. Pistillate inflorescence; F. Extrafloral nectaries along midrib (arrows); G. Staminate flower; H. Pistillate flowers; I. Infructescence with fruits; J. Fruit with sepals persistent (all photos by Nguyen Thi Kim Thanh).

#### Diagnosis.

The new species is similar to *M. leptostachyus* Hook.f. and *M. peltatus* (Geiseler) Müll.Arg. in shrub habit, in having alternate to opposite leaves, indumentum of simple and stellate hairs and yellow glandular scales, leaf margin with denticulate glandular teeth, racemose and unbranched inflorescences. However, it can be fully distinguished from those by the deciduous stipules (vs. present), oblanceolate leaf blade (vs. ovate to obovate in *M. peltatus*, and elliptic in *M. leptostachyus*), penninerved veination (penninerved or palmate in *M. peltatus*, and triplinerved in *M. leptostachyus*), extrafloral nectaries two larger near the base or sometimes 2–6(–8) along midrib (vs. 2–6 small one in *M. peltatus* and two in *M. leptostachyus*), stamens 28–33 (vs. 25–35 in *M. peltatus* and c. 60 in *M. leptostachyus*), pistillate flower with sepal 3 (vs. calyx urceolate, caducous in *M. peltatus*), short style up to 0.4 mm long (vs. 2.8–4.5 mm long in *M. peltatus*, c. 0.2 mm in *M. leptostachyus*), fruits without spines (vs. with spines in *M. peltatus*).

**Figure 2.**
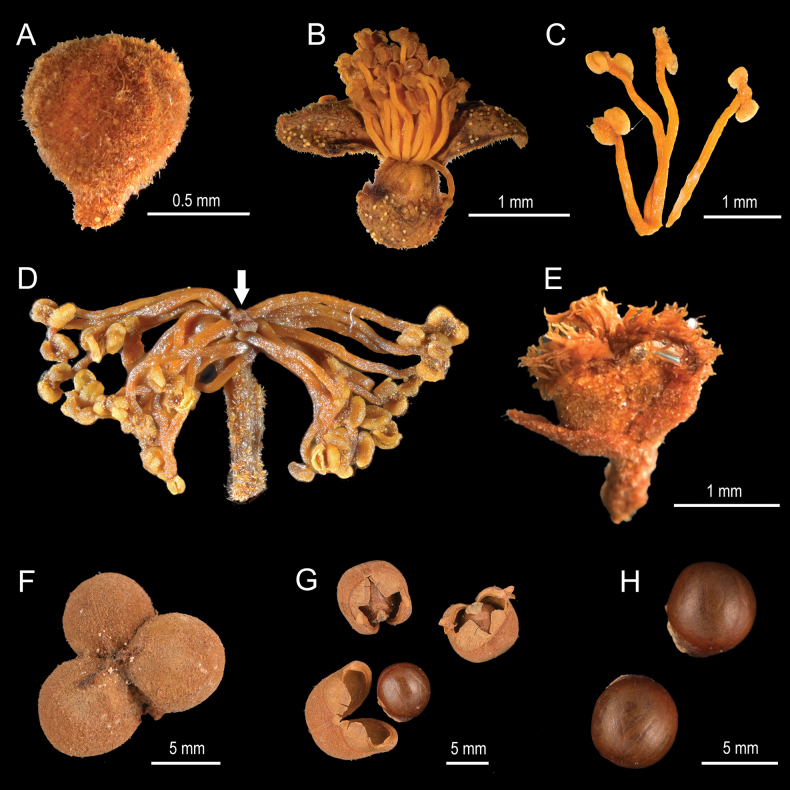
The dried flowers of *Mallotus thinii* Kim Thanh & V.S.Dang. A. Staminate flower in bud; B. Staminate flower when open; C. Stamens with separated thecae; D. Pistillode; E. Pistillate flower; F. Dried fruit without spine; G. Valves and seeds from dehisced capsule; H. Brown seeds (photos taken by Nguyen Thi Kim Thanh and Nguyen Thanh Son).

#### Description.

Dioecious shrubs, rarely monoecious, 0.8–1.5 m tall. Young branches flattened, matured branches roundish with longitudinal ridges. Indumentum pubescent on most young parts and inflorescences, composed of simple and stellate hairs and yellow glandular scales. Stipules subulate, c. 2.2 mm long, pubescent outside, deciduous. Leaves simple, alternate, apically opposite, or whorled; blade oblanceolate to rarely narrow elliptic; 9.7–13.5 × 3.2–4.7 cm; apex bluntly apiculate up to 0.7 cm long; base truncate or slightly cordate, margin dentate for distal 1/2 to 2/3^rd^, with distal-pointing glandular teeth; upper and lower surfaces become glabrous at maturity; venation penninerved, nerves 6–8 per side, lateral vein angle from 25–30°, looped and joined at 1–3 mm from the margin; extrafloral nectaries 2, on basal nerves, elliptic, 1–1.5 × 0.3–0.5 mm, on the upper surface, sometimes 2–6(–8) extra ones, elliptic to orbicular, ca. 0.4 × 0.2–0.4 mm, along midrib, near the base; petiole sulcate, 0.5–3 cm long, covered with stellate hairs. Inflorescences erect, terminal to axillary, unbranched, pubescent with yellow glandular scales, petals, and disc absent. Staminate inflorescence racemes, up to 11 cm long, flowers in groups of up to eight per node; bracts triangular, c. 1 × 0.8 mm. Staminate flowers pedicels 1.3–1.5 mm long; sepal 3–4, free, ovate, c. 1.7 mm long, densely covered with stellate hairs outside and yellow glandular scales outside and inside; stamens 28–33, filaments 1–1.5 mm, thecae two separated by broad connective, ovoid to ellipsoid, 0.2–0.25 × 0.15–0.2 mm, extrorse, opening lengthwise; pistillode present, consisting of 2–3 wart-like appendices. Pistillate inflorescences racemose, up to 9 cm long, bracts triangular, c. 0.8 × 1 mm. Pistillate flowers c. 1.5 mm in diameter; pedicel up to 1 mm long; sepal 3, ovate, ca. 1 mm long, free; ovary 3-locular, hairy, green; style absent to 0.4 mm long; stigmas 3, plumose, up to 1.5 mm long, persistent. Infructescence up to 17 cm long, peduncle 2–3 cm long. Fruits 3-lobed capsules, curved downward when mature, green, subglobose (slightly flattened dorsiventrally), without spines, 0.8–1.2 mm in diameter, 3-locular, densely hairy, and yellow glandular scales, opening into three valves, septicidally and incompletely loculicidally from the base, each valve slightly flattened dorsoventrally, wall ca. 0.7 mm thick. Seeds subglobose, ca. 5 × 4 × 5 mm, ecarunculate, surface smooth, brown.

#### Flowering and fruiting.

Flowering from April to October and fruiting from May to November.

#### Distribution.

**Figure 3.**
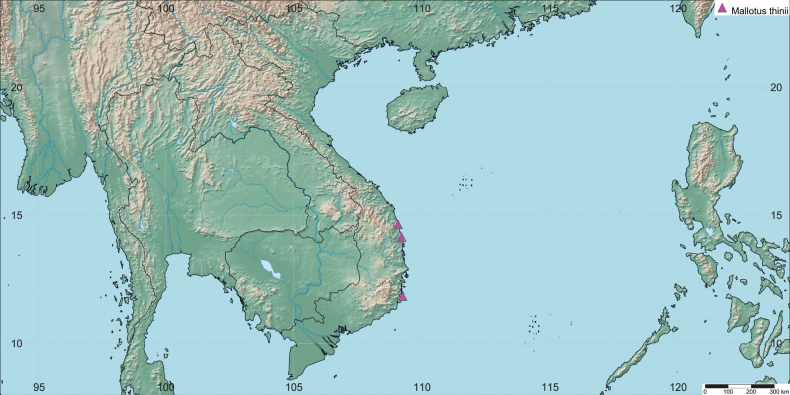
Geographical distribution of *Mallotus thinii* Kim Thanh & V.S.Dang (pink triangles; map created with http://www.simplemappr.net).

This species is known from the coastal areas of south-central Vietnam: Sa Huynh beach in Sa Huynh commune, Quang Ngai province; Dang Vinh Loi beach of Phu My commune, Binh Dinh province; and Binh Ba Island of Nam Cam Ranh commune, Khanh Hoa province (Fig. 3).

#### Habitat.

*Mallotus thinii* grows on sandy soils near the sea, characterized by poor soil fertility, frequent strong sunlight, drought, and wind. This species is distributed at altitudes up to 100 m.

#### Vernacular.

The new species was discovered at three locations, all of which are near the sea. To clarify the difference in habitat with other species within the genus *Mallotus*, we propose the Vietnamese common name “Ruối biển Thìn”.

#### Etymology.

The species is named in honor of Professor Nguyen Nghia Thin from VNU University of Science, who devoted his career to revising the Euphorbiaceae of Vietnam.

#### Uses.

No known uses.

#### Other specimen examined.

Vietnam • Quang Ngai province, Sa Huynh commune, on sandy soils in the local cemetery closed to the beach, 14°38'10.5"N, 109°03'59.5"E, 07 Oct 2018, *Kim Thanh KT181007-12* (HNU!) • Binh Dinh province, Phu My commune, Dang Vinh Loi beach, 14°07'42.3"N, 109°12'42.5"E, 22 Jun 2007, *Thin N.N. NT070622-17* (HNU!) • Khanh Hoa province, Nam Cam Ranh commune, Binh Ba Island, 11°50'07.7"N, 109°14'43.0"E, elevation 100 m, 30 Aug 2023, *Kim Thanh KT230830-04* (HNU!, VNM!).

#### Preliminary conservation status.

Data deficient (DD). *Mallotus thinii* was recorded from three populations in three different provinces, including Quang Ngai, Binh Dinh, and Khanh Hoa. All populations are located outside protected areas and near beaches or tourist areas where there is a high risk of land use change or human impact. At Sa Huynh beach in Quang Ngai, there are only a few restaurants and seafood farms serving tourists, and finally close to Ganh Mountain (14°37'58.9"N, 109°04'06.0"E) is a local cemetery. About 20 individuals were observed. We interviewed local people and learned that there is a larger population near Ganh Mountain. The presence of young individuals in this population is an indicator of the possibility of sexual reproduction process is normal and ongoing. Although we have not had the opportunity to explore that mountain, we believe that the number of individuals in this area is much larger than what we observed. In Dang Vinh Loi beach (Binh Dinh province) and Binh Ba Island (Khanh Hoa province), each population contained approximately 30 individuals. Although these populations are potentially endangered because they are not located in a protected area, from what has been observed and what information is available, this species has adapted very well to the dry conditions of the sand beach and the hot, dry climate of the south central region. According to insufficient information on distribution range and population size, this species can be qualified as Data deficient (DD) (IUCN 2024).

### ﻿A key to the species of *Mallotus* in Vietnam

**Table:** 

1	Leaves opposite, longer than large, pinnatinerved, rarely trinerved or palmate; pistillodes present in male flowers	**2**
–	Leaves large-triangular or rhombic-ovate, alternate, palmately nerved or trinerved; pistillodes absent	**22**
2	Two leaves of each pair similar, not reduced	**3**
–	One leaf of each pair stipule-like	**19**
3	Leaves pinnately nerved or rarely triplinerved with weak basal nerves	**4**
–	Leaves palmatinerved or triplinerved	**10**
4	Inflorescences cauliflorous on the lower stem	***M. phongnhaensis***
–	Inflorescences axillary or terminal	**5**
5	Leaves without puncti-glandules beneath (domatia)	**6**
–	Leaves puncti-glandular beneath	**9**
6	Two black extrafloral nectaries at the leaf base	***M. hanheoensis***
–	Without extrafloral nectaries at the leaf base	**7**
7	Fruits without dense and long spines at the top	***M. poilanei***
–	Fruits sparse and short hairs	**8**
8	Fruits with long and dense hairs; leaf-margins undulate	***M. eberhardtii***
–	Fruits with sparse spines at the top; leaf-margins dentate	***M. sathayensis***
9	Leaves obovate, with six pairs of nerves; male inflorescences few-flowered	***M. yunnanensis***
–	Leaves oblanceolate, with more than eight pairs of nerves; male inflorescences many-flowered	***M. resinosus***
10	Leaves oblong, lanceolate or oblanceolate	**11**
–	Leaves large-ovate, oval or obovate	**15**
11	Leaves weakly triplinerved, glabrous; fruits yellow, glandular with hairs inside the fruit wall	***M. lanceolatus***
–	Leaves not above	**12**
12	Leaves rhombic or lanceolate or oblanceolate, coarsely dentate halfway from apex; leaf lower surface, male and female sepals covered with yellow glandules; tertiary veins parallel and clearly raised below	***M. decipiens***
–	Leaves oblong, long-lanceolate or long-oblanceolate, entire; leaf lower surface, male and female sepals eglandular (except *Mallotus canii*), tertiary veins sparse and not raised beneath	**13**
13	Filaments glabrous; stipules caducous; young branchlets covered with lepidote scales; male buds obovoid	***M. canii***
–	Filaments pubescent; stipules 4–5 mm long; young branchlets glabrous; male buds globose	**14**
14	Male flowers sepals 3, coriaceous, stipules present	***M. glabriusculus***
–	Male flowers sepals 5, chartaceous; stipules deciduous	***M. pierrei***
15	Fruits spinous, whitish stellate hairs	***M. coudercii***
–	Fruit densely echinate	**16**
16	Leaves coriaceous, densely pubescent; fruits slender echinate; pistillodes large	**17**
–	Leaves membranous, glabrous; fruits sparsely strong echinate; pistillodes reduced	**18**
17	Filaments pubescent, sepals 3; stamens 25; pistillode flat-globose	***M. chuyenii***
–	Filaments glabrous, sepals 4–5; stamens 50; pistillode cup-shaped	***M. ustulatus***
18	Male and female flower sepals pubescent; filaments pubescent	***M. glabriusculus***
–	Male and female flower sepals glabrous; filaments glabrous	***M. nanus***
19	Fruit smooth, without spines	***M. ninhthuanensis***
–	Fruit echinate	**20**
20	Reduced leaves suborbicular	***M. vinhhyensis***
–	Reduced leaves narrowly triangular	**21**
21	Non-reduced leaf base rounded to obtuse	***M. hookerianus***
–	Non-reduced leaf base deeply cordate, sometimes half-surrounding the stem	***M. cordatifolius***
22	Fruits smooth; leaves almost epeltate, glandular-granular beneath	**23**
–	Fruits echinate; leaves peltate, rarely epeltate	**29**
23	Ovary 2-locular	***M. repandus***
–	Ovary 3-locular	**24**
24	Leaves large-ovate	**25**
–	Leaves long-ovate to oval or oblanceolate	**26**
25	Fruits ca. 10 mm in diameter, yellow; leaves coriaceous with several extrafloral nectaries marginal in the lower half; climbing shurbs	***M. contubernalis***
–	Fruits ca. 5 mm in diameter, green; leaves chartaceous with extrafloral nectaries at leaf base; erect shrubs	***M. microcapus***
26	Fruit and leaf lower surface covered with red glandular-granules, two extrafloral nectaries next to the basal nerves	***M. philippinensis***
–	Fruit and leaf lower surface not covered with red glandular granules, two extrafloral nectaries on the basal nerves	**27**
27	Indumentum composed of stellate hairs; stipules 3–6 mm long; pistillate flower sepals 4–5; fruits yellowish	***M. pallidus***
–	Indumentum composed of simple and stellate hairs; stipule less than 2.5 mm long; pistillate flower sepals 3; fruits green	**28**
28	Stipules narrowly triangular, caducous; stamens ca. 60	***M. leptostachyus***
–	Stipules subulate, deciduous; stamens 28–33	***M. thinii***
29	Leaves narrowly peltate or epeltate; male flower sepals deciduous; stamens 17–42; fruits less than 1 cm in diameter	**30**
–	Leaves peltate, large ovate; male flower sepals persistent; stamens >45; fruits exceeding 1 cm in diameter	**33**
30	Branchlets and leaves glabrous	***M. floribundus***
–	Branchlets and leaves pubescent	**31**
31	Leaves epeltate	***M. nepalensis***
–	Leaves peltate	**32**
32	Leaves narrow-ovate, not whitish-punctate on the upper surface; margin nearly entire	***M. peltatus***
–	Leaves large-ovate, densely whitish-punctate on the upper surface; margin dentate	***M. thorelii***
33	Fruits sparsely echinate	**34**
–	Fruits densely echinate ± forming a continuous layer	**36**
34	Leaves with brown to yellow beneath	***M. japonicus***
–	Leaves with white beneath	**35**
35	Shrubs or small trees; inflorescences slender, often single or few-branchlets at the basal part, white pubescent; fruits densely whitish echinate	***M. apelta***
–	Trees; inflorescences largely paniculate, brownish pubescent; fruits distantly brown echinate	***M. paniculatus***
36	Leaves subpeltate; fruits robust-shortly echinate	***M. macrostachyus***
–	Leaves peltate; fruits flocculently echinate	**37**
37	Leaves grey-yellow, large peltate, blade loped; indumentum composed of densely floccose-tomentose	***M. barbatus***
–	Leaves narrow peltate, blade entire or rarely lobed, thick and rigid when dry; indumentum composed of red-brown hairs	**38**
38	Infructescence sparsely including fruits with distantly spines	***M. metcalfianus***
–	Infructescence densely including fruits with long straight spines	***M. mollissimus***

## ﻿Discussion

### ﻿Morphological

*Mallotus thinii* occupies a different habitat from other species in Vietnam, namely sandy soils close to the beach. Moreover, it is morphologically distinguished from most of the other previously known *Mallotus* species in Vietnam and nearby areas by the combination of the following morphological features: shrubby habit, deciduous stipules, oblanceolate leaf blades, two larger extrafloral nectaries near the base or sometimes two to four more along the midrib, presence of a pistillode, and fruits without spines. In terms of vegetative morphology, this species could most easily be confused with *M. peltatus* because of its similar leaf arrangement, leaf shape, leaf venation, and extrafloral nectaries (Table 3). However, its female reproductive organs are similar to those of *M. leptostachyus*, which has fruits that are densely hairy and lack spines (Table 3).

**Table 3.** Comparison of *Mallotus thinii* with morphologically most similar species (modified from Gagnepain 1925, Ho 1999, Kiu and Gilbert 2008, and van Welzen and Chayamarit 2020).

Characters	*M. thinii*	*M. leptostachyus*	*M. peltatus*
Habit	shrubs	shrubs to trees	small trees or shrubs
Stipules	deciduous	persistent	persistent
Leaf blades	oblanceolate to rarely narrow elliptic	elliptic	ovate to obovate
Venation	penninerved	tripinerved	penninerved or palmate
Extrafloral nectaries	2 larger glands near the base and sometimes 2–6(–8) more along midrib	2 glands near the base	2–6 small glands near the base
Stamens	28–33	c. 60	25–35
Pistillode	present	present	absent
Pistillate flower	sepals 3, style absent to 0.4 mm long	sepals 3, style c. 0.2 mm long	calyx urceolate, caducous, style 2.8–4.5 mm long
Fruit	without spines, densely hairy	without spines, densely hairy	with spines, subglabrous
Seeds	subglobose, brown	–	subglobose, brown

The new species described here should be classified in Mallotus Lour. sect. Philippinenses Pax & K.Hoffm., based on shared stellate and simple hairs, yellow glandular scales on most parts, alternate leaves, blades that are not peltate with two basal extrafloral nectaries on the upper surface, unisexual inflorescences, and fruits lacking spines (Sierra et al. 2010). Phylogenetic evidence has indicated that sect. Philippinenses, as presently circumscribed, is not monophyletic (Sierra et al. 2010), and the new species would fall into the “Philippinenses grade,” which comprises basal branches leading to an embedded clade of sect. Mallotus.

### ﻿Phylogenetic results

**Figure 4.**
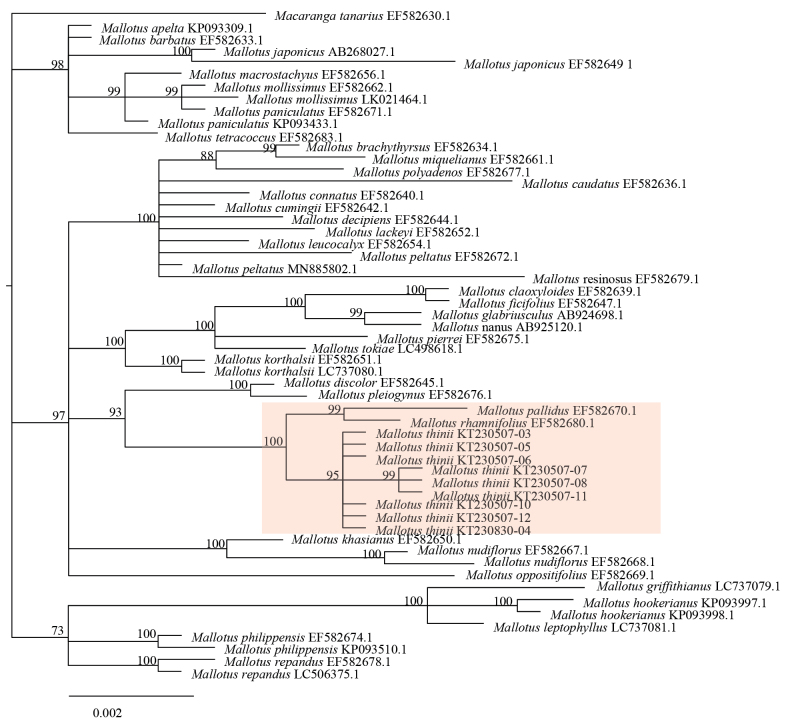
Bayesian phylogram based on *matK* sequences.

**Figure 5.**
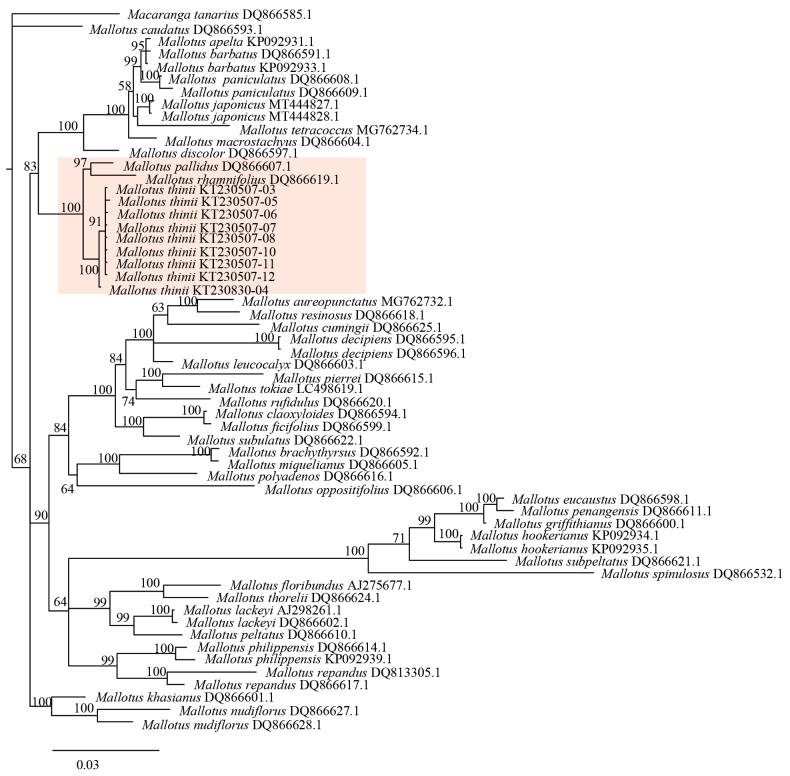
Bayesian phylogram based on ITS sequences.

**Figure 6.**
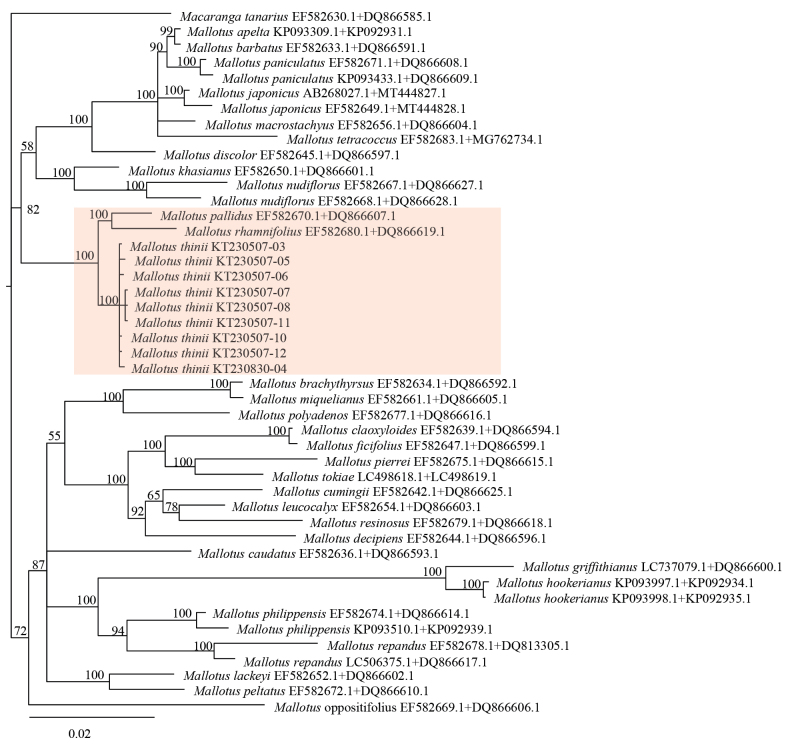
Bayesian phylogram based on combined *matK* and ITS sequences (numbers above branches are Bayesian posterior probabilities). *Macaranga tanarius* sequences served as an outgroup.

Phylogenetic analyses of *matK* and ITS sequence datasets, either separately or in combination, all resolved the nine samples of the new species *Mallotus thinii* as a monophyletic clade (BP = 100) distinct from morphologically similar species such as *M. peltatus* (Figs 4–6). *M. thinii* forms a sister clade to the one containing *M. pallidus* and *M. rhamnifolius* (Figs 4–6). However, because of the limited *Mallotus* matK and ITS sequences available in GenBank, only 30 other *Mallotus* species were included in the phylogenetic analyses. Therefore, the sister species to *M. thinii* remains uncertain, and more extensive taxonomic sampling is needed to clarify its evolutionary origin.

## Supplementary Material

XML Treatment for Mallotus thinii

## ﻿Additional information

### Use of AI

No use of AI was reported.

### Author ORCIDs

Nguyen Thi Kim Thanh https://orcid.org/0000-0003-2948-6385

Nguyen Diep Anh https://orcid.org/0009-0000-5370-6048

Tran Thi Thuy Anh https://orcid.org/0009-0004-6170-4666

La Thi Thuy https://orcid.org/0009-0007-8784-4597

Nguyen Anh Duc https://orcid.org/0009-0008-8938-1343

Tran Duc Long https://orcid.org/0000-0001-9232-0244

Van-Son Dang https://orcid.org/0000-0001-8681-4141

### Data availability

All data supporting the findings of this study are available in a public database (GenBank), as specified in the main text.
